# Pseudomonas lyxosi sp. nov., Pseudomonas arabinosi sp. nov. and Pseudomonas frigoris sp. nov., isolated from glaciers

**DOI:** 10.1099/ijsem.0.006799

**Published:** 2025-06-06

**Authors:** Chan Zhao, Yu-Hua Xin, Qing Liu

**Affiliations:** 1 State Key Laboratory of Animal Biotech Breeding and College of Biological Sciences, China Agricultural University, Beijing 100193, P.R. China; 2 China General Microbiological Culture Collection Center, Institute of Microbiology, Chinese Academy of Sciences, Beijing 100101, P.R. China; 3 Beijing Key Laboratory of Genetic Element Biosourcing & Intelligent Design for Biomanufacturing, Beijing 100101, P.R. China

**Keywords:** glacier, phylogenomic, *Pseudomonas*

## Abstract

Three Gram-stain-negative, rod-shaped, motile bacterial strains, each with a single polar flagellum, designated LB3P38^T^, LT1P18^T^ and ZB1P45^T^, were isolated from glacier samples on the Tibetan Plateau, P.R. China. These strains grew at temperatures ranging from −2 to 32 °C (optimum 25–30 °C) and at pH values of 5.0–11.0 (optimum pH 7.0). The similarities of 16S rRNA gene sequences among these strains ranged from 99.67% to 99.93%. Phylogenetic analysis based on 16S rRNA gene sequences confirmed their affiliation with the genus *Pseudomonas*. Phylogenomic analysis positioned these strains in close relation to *Pseudomonas svalbardensis* PMCC 200367^T^ and *Pseudomonas frederiksbergensis* LMG 19851^T^. Average nucleotide identity values and digital DNA–DNA hybridization values between these strains and other type strains of the genus *Pseudomonas* were below 94.6% and 63.4%, respectively. The predominant fatty acids identified in these strains were C_16:0_, C_17:0_ cyclo and summed feature 3 (C_16:1_ *ω7*c and/or C_16:1_ *ω6*c). Based on the combined phenotypic and phylogenetic evidence, each strain is proposed to represent a novel species within the genus *Pseudomonas*, with the names *Pseudomonas lyxosi* sp. nov. (type strain=LB3 P38^T^=CGMCC 1.11284^T^=JCM 37126^T^), *Pseudomonas arabinosi* sp. nov. (type strain=LT1 P18^T^=CGMCC 1.11310^T^=JCM 37131^T^) and *Pseudomonas frigoris* sp. nov. (type strain=ZB1 P45^T^=CGMCC 1.23235^T^=JCM 37141^T^) proposed.

The genus *Pseudomonas*, belonging to the family *Pseudomonadaceae*, was originally described by Migula [1], with *Pseudomonas aeruginosa* as the type species. Most *Pseudomonas* strains are Gram-negative, aerobic and rod-shaped. *Pseudomonas* species exhibit extensive genetic diversity and remarkable metabolic versatility [2,3]. At the time of writing, the genus *Pseudomonas* includes over 350 species with validly published names [4]. Hesse *et al*. [5] established a genomic framework for *Pseudomonas* evolutionary history, identifying 13 evolutionary groups by comparing genomes from type strains of 163 species and 3 subspecies. *Pseudomonas* is widely distributed across diverse habitats, including plants [6], soil [7], freshwater [8], marine environments [9] and glaciers [10]. Certain species, such as *P. aeruginosa* and *Pseudomonas syringae*, are known as human and plant pathogens, respectively [11]. Studies have shown that *Pseudomonas* is one of the most dominant bacterial genera present in glacial soils and snow [10]. In this study, three *Pseudomonas* strains, LB3P38^T^, LT1P18^T^ and ZB1P45^T^, were isolated from glacier samples in P.R. China and characterized using a polyphasic taxonomic approach. The results indicated that these strains represent three novel species, proposed here as *Pseudomonas lyxosi* sp. nov., *Pseudomonas arabinosi* sp. nov. and *Pseudomonas frigoris* sp. nov., respectively.

## Isolation and ecology

Strains LB3P38^T^ and LT1P18^T^ were isolated from ice and cryoconite samples, respectively, collected from the Laigu Glacier (29.3087826° N, 96.8186951° E), and strain ZB1P45^T^ was isolated from an ice sample from the Zepu Glacier (30.276556° N, 95.2508392°E), both on the Tibetan Plateau, P.R. China, in October 2016. The samples were homogenized, serially diluted with sterile water and plated onto peptone, yeast extract and glucose agar (PYG, 0.5% bacto peptone (Difco), 0.02% yeast extract, 0.5% glucose, 0.3% beef extract, 0.05% NaCl, 0.15% MgSO_4_·7H_2_O, pH adjusted to 7.0). Following incubation at 14 °C for 15 days, strains LB3P38^T^, LT1P18^T^ and ZB1P45^T^ were isolated and purified by repeated streaking on PYG agar. Strains were routinely cultured on Nutrient Agar (NA) at 14 °C and preserved in 10% (v/v) glycerol suspensions in a liquid nitrogen storage tank. Strain *Pseudomonas svalbardensis* CCTCC AB 2023225^T^, which is most closely related to the three strains obtained from the China Center for Type Culture Collection, was used as an experimental control.

## 16s rRNA phylogeny

Genomic DNA was extracted using the TaKaRa MiniBEST Bacteria Genomic DNA Extraction Kit Ver. 3.0 (TaKaRa, Dalian, China) following the manufacturer’s instructions. The 16S rRNA gene was amplified and sequenced using the bacterial universal primers 27F and 1492R [11]. Complete 16S rRNA gene sequences were also extracted from the genome assemblies of the three strains. These sequences were compared using the EzBioCloud database [12] and BLAST +programme V2.14 [13]. 16S rRNA gene sequences of closely related taxa were retrieved for phylogenetic analysis. Multiple sequence alignments were performed using the clustal_W program implemented in mega7 [14]. Phylogenetic trees were reconstructed using the neighbour-joining (NJ) [15] method in mega7, with genetic distances calculated using Kimura’s two-parameter model [16]. Tree topologies were evaluated with 1,000 bootstrap replicates [17].

Nearly complete 16S rRNA gene sequences of strains LB3P38^T^ (1,412 bp), LT1P18^T^ (1,328 bp) and ZB1P45^T^ (1,374 bp) were identical to the corresponding complete sequences (1,534 bp) retrieved from their genomic sequence. Sequence similarities among the three strains ranged from 99.67% to 99.93%. Blastn and EzBioCloud analyses confirmed that the three strains belong to the genus *Pseudomonas*. Type strains with 16S rRNA gene sequence similarities >98.6% to the three strains are listed in Table S1, available in the online Supplementary Material. Strains LB3P38^T^ and LT1P18^T^ shared the highest sequence similarity with * P. nunensis* In5T (99.66%), followed by *P. silesiensis* A3^T^ (99.52%), *P. piscicola* P50^T^ (99.51%) and *P. mandelii* NBRC 103147^T^ (99.45%). Strain ZB1P45^T^ showed the highest sequence similarity with *P. nunensis* In5^T^ (99.93%), followed by *P. silesiensis* A3^T^ (99.79%), * P. mandelii* NBRC 103147^T^ (99.73%), *P. farris* SWRI79^T^ (99.73%), *P. svalbardensis* S025^T^ (99.73%) and *P. frederiksbergensis* JAJ28^T^ (99.73%). The NJ tree showed that strains LB3P38^T^ and LT1P18^T^ formed a distinct clade, separated from other species with 99% bootstrap support, whereas strain ZB1P45^T^ clustered with *P. svalbardensis* S025^T^ and *P. nunensis* In5^T^ with less than 50% bootstrap support (Fig. S1).

## Genome features

Genomic DNA was sequenced using a combination of long-read shotgun sequencing with the PacBio Sequel II (Pacific Biosciences, CA, USA) and short-read sequencing via Illumina sequencing platforms. Short-reads were generated using the Illumina Hiseq 4000 platform (Illumina, San Diego, CA, USA) with 150 bp paired-end reads according to the manufacturer’s protocols. Complete genome sequences were assembled and polished using Unicycler v0.5.1, integrating both short-reads and long-reads [18]. Genome sequence quality was evaluated based on completeness and contamination rates using CheckM2 v1.0.1 [19] and QUAST v5.2 [20]. The genomes of the three strains were annotated using Bakta version 1.7.0 [21]. Maximum-likelihood (ML) phylogenomic trees were constructed with the IQ-TREE program v2.0.7 [22] using concatenated alignment of 81 core genes extracted via the UBCG2 pipeline [23]. The best-fit nucleotide substitution model (GTR+F+I+R9) was applied, with topologies supported by 1,000 bootstrap replicates. Average nucleotide identity (ANI) values were calculated using the FastANI program [24]. Digital DNA–DNA hybridization (dDDH) values were determined using the Type (Strain) Genome Server [25].

**Fig. 1.**
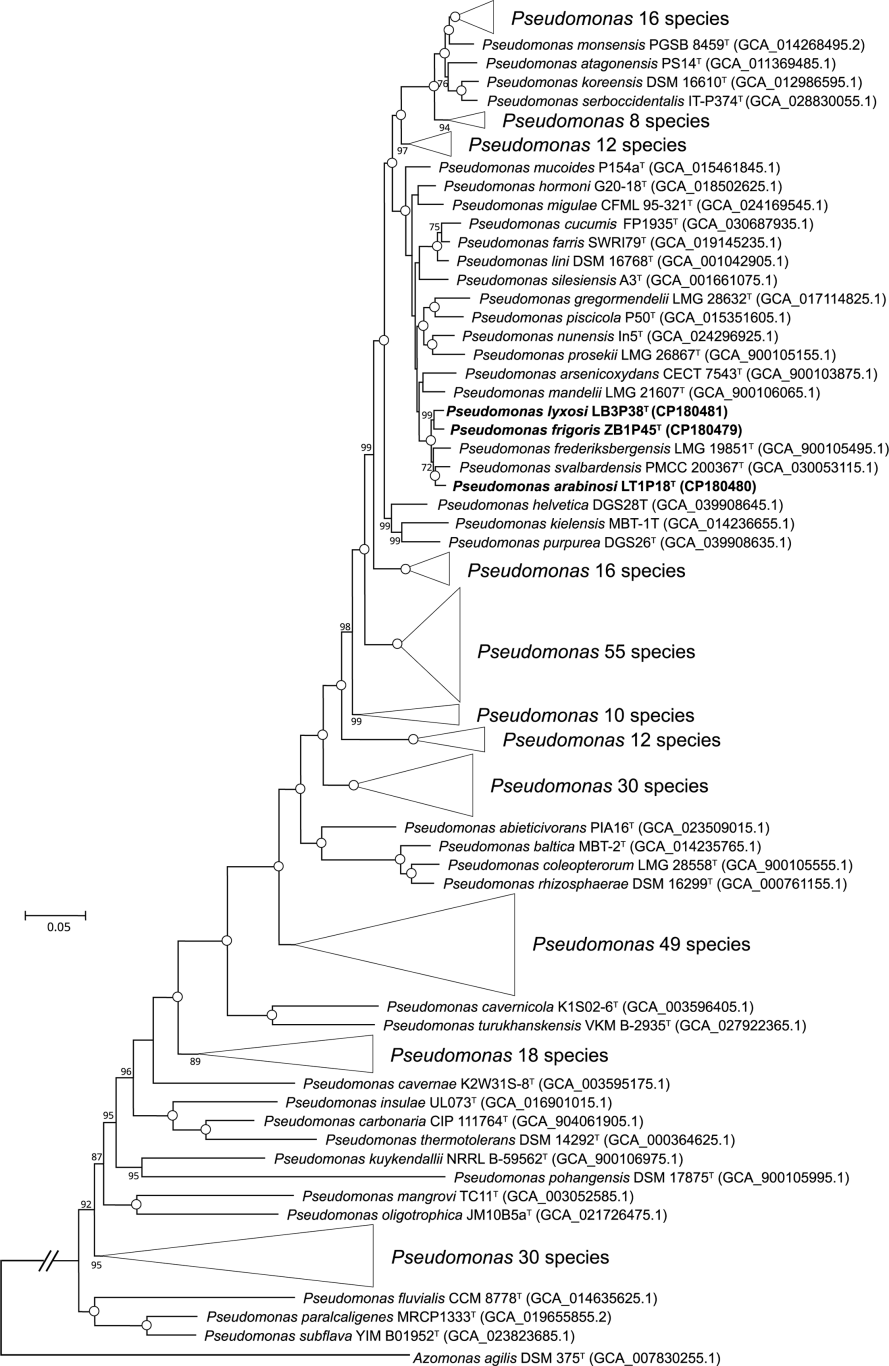
Phylogenetic tree of strains LB3P38^T^, LT1P18^T^, ZB1P45^T^ and related taxa inferred using ML algorithms in IQ-TREE based on the concatenated alignment of 81 core genes. Bootstrap values (>70%) based on 1,000 replicates are shown at the branch nodes. Open circles indicate nodes with 100% bootstrap support. *Azomonas agilis* DSM 375^T^ was used as an outgroup. Scale bar, 0.05 substitutions per nucleotide position.

Assembled genome information of the three strains is presented in Table S2. The genomes of strains LB3P38^T^, LT1P18^T^ and ZB1P45^T^ were circular and complete, with CheckM2 confirming 100% completeness and contamination levels of 1.38%, 1.63% and 1.33%, respectively. The G+C content was 58.9% for LB3P38^T^, 58.8% for LT1P18^T^ and 58.7% for ZB1P45^T^. To determine their taxonomic status, 295 genomic sequences of *Pseudomonas* species with validly published names were downloaded for phylogenomic analysis. The phylogenomic tree, constructed from core gene alignments, showed that strains LT1P18^T^, LB3P38^T^, ZB1P45^T^, *P. svalbardensis* PMCC 200367^T^ and *P. frederiksbergensis* LMG 19851^T^ formed a distinct lineage with 100% bootstrap support. Within this lineage, LB3P38^T^ and ZB1P45^T^ clustered together, while LT1P18^T^ grouped with *P. svalbardensis* PMCC 200367^T^ (Fig. 1). Based on the taxonomic framework established by Garrido-Sanz *et al*. [26] and expanded by Hesse *et al*. [5], these strains were classified within the *P. fluorescens* group, specifically the *P. mandelii* subgroup, as supported by their close phylogenetic relationship with *P. mandelii* LMG 21607^T^ in the phylogenomic tree.

The ANI and dDDH values between strains LT1P18^T^, LB3P38^T^, ZB1P45^T^, *P. svalbardensis* PMCC 200367^T^ and *P. frederiksbergensis* LMG 19851^T^ were 93.0–94.6% and 52.1–63.4%, respectively (Table S3). Additionally, ANI and dDDH values between the three strains and other *Pseudomonas* species with validly published names, for which genome sequences were available from NCBI, were also calculated. All resulting ANI and dDDH values were below 89.4% and 37.8%, respectively. These values align with standard criteria for delineating distinct species (ANI <95–96% and dDDH <70%) [27,28], supporting the classification of strains LT1P18^T^, LB3P38^T^ and ZB1P45^T^ as three novel species within the genus *Pseudomonas*.

## Physiology and chemotaxonomy

Colony morphology of the three novel strains was observed on NA plates. Gram staining was performed as described previously [29]. Cell morphology was examined using a JEM-1400 transmission electron microscopy (JEOL Ltd., Tokyo, Japan). Motility was observed by oil-immersion phase-contrast microscopy using the hanging drop method. Growth at various temperatures (−2–42 °C) was evaluated in Nutrient Broth (NB) medium. The pH range for growth was assessed in NB medium adjusted to pH 4.0–11.0 (at 1.0 pH unit intervals), using appropriate biological buffers (0.2 M Na_2_HPO_4_/NaH_2_PO_4_·2H_2_O for pH 5–8 and 0.2 M Na_2_CO_3_/NaHCO_3_ for pH 9–10). NaCl tolerance (0–6%, w/v, at 0.5% intervals) was tested in NB medium. Anaerobic growth was evaluated in NB supplemented with 1% (w/v) KNO₃ in anaerobic culture tubes. The capacity to hydrolyse starch, casein, tributyrin and Tween 80 was tested following Smibert and Krieg using NA plate as the basal medium [30]. Catalase activity was determined using bubble production in 3% (v/v) H_2_O_2_, while cytochrome oxidase activity was assessed using 1% (w/v) tetramethyl-p-phenylenediamine. Carbon source utilization was tested using API 50 CH strips (bioMérieux) with a basal medium (0.2% (NH_4_)_2_SO_4_, 0.05% NaH_2_PO4·H_2_O, 0.05% K_2_HPO4, 0.02% MgSO_4_·7H_2_O and 0.01 % CaCl_2_·2H_2_O). Additional biochemical tests were performed using API 20NE, API 20E and API ZYM strips (bioMérieux) according to the manufacturer’s instructions. For cellular fatty acids analysis, cell masses were harvested from the NA plate after incubation at their optimal temperature of 28 °C for 1 day. Fatty acids were extracted following the standard protocol of the MIDI 6.0 system (MIDI Inc., Newark, DE, USA) and analysed using the 6890 N Gas Chromatograph (Agilent Technologies, Santa Clara, CA, USA) with the Sherlock Microbial Identification System version 6.0 [31].

Strains LB3P38^T^, LT1P18^T^ and ZB1P45^T^ were Gram-stain-negative, rod-shaped bacteria, each possessing a single flagellum (Fig. S2). These strains grew at −2–32 °C, pH 5.0–11.0. All strains were positive for oxidase and catalase activities. Strains LB3P38^T^ and LT1P18^T^ grew at NaCl concentrations of 0–3.5% (w/v), exhibited weak growth at 4.0 and 5.0% NaCl (w/v), but failed to grow at 6.0% NaCl (w/v). Strain ZB1P45^T^ grew at 0–3.5% NaCl (w/v), showed weak growth at 4.0% (w/v), but could not grow at 5.0% (w/v). Nitrate reduction was positive for all three strains; strains LB3P38^T^ and ZB1P45^T^ reduced nitrates to nitrites, while strain LT1P18^T^ reduced nitrates to nitrogen. Detailed phenotypic characteristics of the three novel strains are provided in the species description. Distinguished phenotypic traits between strains LB3P38^T^, LT1P18^T^, ZB1P45^T^ and the reference strain are listed in Table 1.

**Table 1.** Differential characteristic phenotype of strains LB3P38^T^, LT1P18^T^, ZB1P45^T^ and their closest relative *P. svalbardensis* CCTCC AB 2023225^T^

Characteristic	1	2	3	4
Temperature range for growth (℃)	−2–32	−2–32	−2–32	4–30*
NaCl range for growth (%, w/v)	0–5.0	0–5	0–4	0–7*
pH range for growth	5–11	5–11	5–11	4–9.5*
Reduction of nitrates to nitrogen	−	+	−	+
Aesculin hydrolysis	−	−	−	＋
Gelatin hydrolysis	＋	＋	−	＋
**Enzyme activity:**				
Trypsin	−	＋	＋	＋
α-Chymotrypsin	−	−	＋	＋
Urease	＋	−	＋	＋
Tryptophan deaminase	−	−	−	＋
**Utilization of:**				
d-Arabinose	−	＋	−	−
Sorbitol	−	−	−	＋
*N*-acetylglucosamine	＋	＋	＋	−
Esculin	−	−	−	＋
Salicin	−	−	−	＋
D-Cellobiose	−	−	−	＋
D-Maltose	−	−	−	＋
D-Melibiose	−	−	−	＋
D-Trehalose	＋	＋	−	＋
Starch	−	−	−	＋
Glycogen	−	−	−	＋
d-Lyxose	＋	−	−	−
d-Tagatose	−	−	−	＋
l-Fucose	−	＋	−	＋
5-Ketogluconate	−	−	−	＋

Strains: 1, LB3P38T; 2, LT1P18T; 3, ZB1P45T; 4, *P. svalbardensis* CCTCC AB 2023225T. +, Positive; –, negative.

*Data were obtained from Ge *et al*. [7].

The cellular fatty acid profiles of three novel strains were analysed and compared with those of the reference strains *P. svalbardensis* CCTCC AB 2023225^T^ and *P. frederiksbergensis* LMG 19851^T^, as shown in Table 2. All four strains contained C_16:0_ and summed feature 3 (C_16:1_ *ω7*c and/or C_16:1_ *ω6*c) as their predominant fatty acid components. However, distinct variations were observed in the proportions of specific fatty acids across the strains. The novel strains and *P. frederiksbergensis* LMG 19851^T^ displayed significantly higher levels of C_17:0_ cyclo, ranging from 10.0% to 19.7%, compared with *P. svalbardensis* CCTCC AB 2023225^T^ (<1.0%). Additionally, hydroxylated fatty acids were more abundant in the three novel strains than in *P. svalbardensis* CCTCC AB 2023225^T^. In particular, C_10:0_ 2-OH and C_12:0_ 3-OH were detected at 3.8–7.0% in the novel strains but were absent in *P. svalbardensis* CCTCC AB 2023225^T^. Moreover, strain LB3P38^T^ was uniquely distinguished by the presence of C_12:1_ 3-OH at 4.7%, a fatty acid not observed in the other strains.

**Table 2.** Cellular fatty acid compositions (%) of strains LB3P38^T^, LT1P18^T^, ZB1P45^T^ and their closest relatives

Fatty acid	1	2	3	4	5*
**Saturated**					
C_12:0_	3.1	2.9	2.7	5.23	4.7
C_14:0_	–	–	–	tr	tr
C_16:0_	28.9	29.0	31.6	32.14	32.0
C_18:0_	tr	tr	1.7	tr	tr
C_17:0_ cyclo	10.0	17.0	16.2	tr	19.7
**Unsaturated**					
C_14:1_ *ω5*c	–	–	–	tr	–
**Branched**					
*Anteiso*-C_14:0_	–	–	–	1.13	–
*Anteiso*-C_15:0_	–	tr	tr	tr	–
**Hydroxy**					
C_10:0_ 3-OH	6.8	5.9	4.0	–	3.7
C_12:0_ 2-OH	4.9	4.7	4.2	3.32	–
C_12:0_ 3-OH	7.0	5.3	3.8	–	3.3
C_12:1_ 3-OH	4.7	–	tr	–	tr
Summed feature*					
3	22.3	17.1	19.2	38.63	23.2
8	8.5	13.7	13.0	9.34	7.0

Strains: 1, LB3P38T; 2, LT1P18T; 3, ZB1P45T; 4, *P. svalbardensis* CCTCC AB 2023225T; 5, *P. frederiksbergensis* LMG 19851T. Values are percentages of the total fatty acids. TR, traces (less than 1% of the total fatty acids); −, not detected. *Summed Features are fatty acids that cannot be resolved reliably from another fatty acid using the chromatographic conditions chosen. The MIDI system groups these fatty acids together as one feature with a single percentage of the total. Summed features consist of: 3, C_16:1_ *ω7*c*/*C1_6:1_ *ω6*c; 8, C_18:1_ *ω6*c*/*C1_8:1_ *ω7*c.

*Data were obtained from Ge *et al*. [7].

Based on their phylogenetic, physiological, chemotaxonomic and genotypic features, strains LB3P38^T^, LT1P18^T^ and ZB1P45^T^ are proposed to represent three novel species of the genus *Pseudomonas*, for which the names *P. lyxosi* (LB3P38^T^=CGMCC 1.11284^T^=JCM 37126^T^) sp. nov., *P. arabinosi* (LT1P18^T^=CGMCC 1.11310^T^=JCM 37131^T^) sp. nov. and *P. frigoris* sp. nov. (ZB1P45^T^ = CGMCC 1.23235^T^ = JCM 37141^T^) are proposed.

## Description of *Pseudomonas lyxosi* sp. nov.

*Pseudomonas lyxosi* (ly.xo’si. N.L. gen. n. *lyxosi*, of lyxose).

Cells are Gram-stain-negative, aerobic, motile by means of a monopolar flagellum. 1.1–1.7 µm in length and 0.8–1.0 µm in width. Colonies are 1–3 mm in diameter, round and pale yellow after 1–2 days of incubation at 28 °C on NA plate. Growth occurs at −2–32 °C (optimum, 25–30 °C), pH 5.0–11.0 (optimum, pH 7.0) and 0–5.0% (w/v) NaCl (optimum, 1%). Positive for oxidase and catalase. Hydrolyse casein, gelatin, starch and tributyrin, but not esculin and Tween 80. Positive for reduction of nitrates to nitrites, citrate utilization, urease, Voges–Proskauer test, arginine dihydrolase, lysine decarboxylase, ornithine decarboxylase, alkaline phosphatase, esterase (C4), esterase lipase (C8), lipase (C14), leucine arylamidase, valine arylamidase, naphthol-AS-BI-phosphohydrolase and acid phosphatase. Negative for H_2_S production, indole production, fermentation of glucose, *β*-galactosidase, cystine arylamidase, trypsin, α-chymotrypsin, *α*-galactosidase, *β*-glucuronidase, α-glucosidase, β-glucosidase, *N*-acetyl-*β*-glucosaminidase, *α*-mannosidase and *α*-fucosidase. Acids are produced from d-glucose, l-rhamnose, sucrose, melibiose, amygdalin and l-arabinose. No acids are produced from d-manitol, inositol and sorbitol. Utilizes the following carbohydrate as the sole carbon source: glycerol, l-arabinose, d-ribose, d-galactose, d-glucose, d-fructose, d-mannitol, inositol, d-mannitol, *N*-acetylglucosamine, sucrose, trehalose, d-lyxose, d-arabitol, gluconate and 2-ketogluconate. Cannot utilize the following carbohydrates: erythritol, d-arabinose, d-xylose, l-xylose, d-adonitol, methyl-*β*-d-xylopyranoside, l-sorbose, l-rhamnose, dulcitol, d-sorbitol, methyl-*α*-d-mannopyranoside, methyl-*α*-d-glucopyranoside, amygdalin, arbutin, esculin, salicin, cellobiose, maltose, lactose, melibiose, inulin, melezitose, raffinose, starch, glycogen, xylitol, gentiobiose, turanose, d-tagatose, d-fucose, l-fucose, l-arabitol and 5-ketogluconate. The major cellular fatty acids are C_16:0_, summed feature 3 (C_16:1_ *ω7*c and/or C_16:1_ *ω6*c) and C_17:0_ cyclo. The genomic DNA G+C content is 58.9 mol%.

The type strain LB3P38^T^ (=CGMCC 1.11284^T^=JCM 37126^T^) was isolated from an ice sample collected from the Laigu Glacier on the Tibetan Plateau, P.R. China. The GenBank accession no. for the 16S rRNA gene sequence reported in this paper is PQ844665. The genome sequence has been deposited at DDBJ/ENA/GenBank under the accession no. CP180481.

## Description of *Pseudomonas arabinosi* sp. nov.

*Pseudomonas arabinosi* (a.ra.bi.no’si. N.L. gen. n. *arabinosi*, pertaining to arabinose.)

Cells are Gram-stain-negative, aerobic, motile by means of a monopolar flagellum. 1.7–2.4 µm in length and 0.9–1.1 µm in width. Colonies are 1–2 mm in diameter, round and pale yellow after 1–2 days of incubation at 28 °C on NA plate. Growth occurs at −2–32 °C (optimum, 25–30 °C), pH 5.0–11.0 (optimum, pH 7.0) and 0–5.0% (w/v) NaCl (optimum, 1%). Positive for oxidase and catalase. Hydrolyse casein, gelatin, starch and tributyrin, but not esculin and Tween 80. Positive for reduction of nitrates to nitrogen, citrate utilization, Voges–Proskauer test, arginine dihydrolase, lysine decarboxylase, ornithine decarboxylase, alkaline phosphatase, esterase (C4), esterase lipase (C8), lipase (C14), leucine arylamidase, valine arylamidase, cystine arylamidase, naphthol-AS-BI-phosphohydrolase and acid phosphatase. Negative for H_2_S production, indole production, fermentation of glucose, *β*-galactosidase, trypsin, *α*-chymotrypsin, *α*-galactosidase, *β*-glucuronidase, *α*-glucosidase, *β*-glucosidase, *N*-acetyl-*β*-glucosaminidase, *α*-mannosidase and *α*-fucosidase. Acids are produced from l-rhamnose, sucrose, melibiose, amygdalin, and l-arabinose. No acids are produced from d-glucose, d-manitol, inositol and sorbitol. Utilizes the following carbohydrate as the sole carbon source: glycerol, d-arabinose, l-arabinose, d-ribose, d-galactose, d-glucose, d-fructose, d-mannose, inositol, d-mannitol, *N*-acetylglucosamine, sucrose, trehalose, l-fucose, d-arabitol, gluconate and 2-ketogluconate. Cannot utilize the following carbohydrates: erythritol, d-xylose, l-xylose, d-adonitol, methyl-β-d-xylopyranoside, l-sorbose, l-rhamnose, dulcitol, d-sorbitol, methyl-α-d-mannopyranoside, methyl-α-d-glucopyranoside, amygdaline, arbutin, esculin, salicin, cellobiose, maltose, lactose, melibiose, inulin, melezitose, raffinose, starch, glycogene, xylitol, gentiobiose, turanose, d-lyxose, d-tagatose, d-fucose, l-arabitol and 5-ketogluconate. The major cellular fatty acids are C_16:0_, summed feature 3 (C_16:1_ *ω7*c and/or C_16:1_ *ω6*c), C_17:0_ cyclo and summed feature 8 (C_18:1_ *ω*7*c* and/or C_18:1_ *ω*6*c*). The genomic DNA G+C content is 58.8 mol%.

The type strain LT1P18^T^ (=CGMCC 1.11310^T^=JCM 37131^T^) was isolated from a cryoconite sample collected from the Laigu Glacier on the Tibetan Plateau in the southwest of China. The GenBank accession number for the 16S rRNA gene sequence reported in this paper is PQ844666. The genome sequence has been deposited at DDBJ/ENA/GenBank under the accession no. CP180480.

## Description of *Pseudomonas frigoris* sp. nov.

*Pseudomonas frigoris* (fri’go.ris. L. gen. n. *frigoris*, of the cold).

Cells are Gram-stain-negative, facultatively anaerobic, motile by means of a monopolar flagellum. 1.4–3.0 µm in length and 0.9–1.2 µm in width. Colonies are 1–3 mm in diameter, round and pale yellow after 1–2 days of incubation at 28 °C on NA plate. Growth occurs at −2–32 °C (optimum, 25–30 °C), at pH 5.0–11.0 (optimum, pH 7.0) and 0–4.0% (w/v) NaCl (optimum, 1%). Positive for oxidase and catalase. Hydrolyse casein, gelatin, starch and tributyrin, but not esculin and Tween 80. Positive for reduction of nitrates to nitrites, citrate utilization, urease, Voges–Proskauer test, arginine dihydrolase, lysine decarboxylase, ornithine decarboxylase, alkaline phosphatase, esterase (C4), esterase lipase (C8), lipase (C14), leucine arylamidase, valine arylamidase, cystine arylamidase, trypsin, naphthol-AS-BI-phosphohydrolase and acid phosphatase. Negative for H_2_S production, indole production, fermentation of glucose, *β*-galactosidase, *α*-chymotrypsin, *α*-galactosidase, *β*-glucuronidase, *α*-glucosidase, *β*-glucosidase, *N*-acetyl-*β*-glucosaminidase, *α*-mannosidase and *α*-fucosidase. Acids are produced from l-rhamnose and melibiose. No acids are produced from d-glucose, d-manitol, inositol, sorbitol, sucrose, amygdalin and l-arabinose. Utilizes the following carbohydrate as the sole carbon source: glycerol, l-arabinose, d-ribose, d-galactose, d-glucose, d-fructose, d-mannose, inositol, d-mannitol, *N*-acetylglucosamine, sucrose, d-arabitol, gluconate and 2-ketogluconate. Cannot utilize the following carbohydrates: erythritol, d-arabinose, d-xylose, l-xylose, d-adonitol, methyl-β-d-xylopyranoside, l-sorbose, l-rhamnose, dulcitol, d-sorbitol, methyl-α-d-mannopyranoside, methyl-α-d-glucopyranoside, amygdaline, arbutin, esculin, salicin, cellobiose, maltose, lactose, melibiose, trehalose, inulin, melezitose, raffinose, starch, glycogene, xylitol, gentiobiose, turanose, d-lyxose, d-tagatose, d-fucose, l-fucose, l-arabitol and 5-ketogluconate. The major cellular fatty acids are C_16:0_, summed feature 3 (comprising C_16:1_ *ω7*c and/or C_16:1_ *ω6*c), C_17:0_ cyclo and summed feature 8 (comprising C_18:1_ *ω*7*c* and/or C_18:1_ *ω*6*c*). The genomic DNA G+C content is 58.7 mol%.

The type strain (strain ZB1P45^T^=CGMCC 1.23235^T^=JCM 37141^T^) was isolated from an ice sample of Zepu Glacier in Tibet Autonomous Region, China (30.276556 N, 95.2508392 E). The GenBank accession number for the 16S rRNA gene sequence of strain ZB1P45^T^ is PQ844667. The genome sequence of strain ZB1P45^T^ has been deposited at DDBJ/ENA/GenBank under the accession no. CP180479.

## Supplementary material

10.1099/ijsem.0.006799 Uncited Supplementary Material 1.

## Abbreviations

ANI: average nucleotide identity
dDDH: digital DNA–DNA hybridization
ML: maximum-likelihood
NA: Nutrient Agar
NB: Nutrient Broth
NJ: neighbour-joining
PYG: peptone, yeast extract and glucose
