# Supplementary material for: Pseudomonas lyxosi sp. nov., Pseudomonas arabinosi sp. nov. and Pseudomonas frigoris sp. nov., isolated from glaciers
Source: Int J Syst Evol Microbiol. 2025 Jun 6;75(6):006799. doi: 10.1099/ijsem.0.006799 (PMC12144318; doi:10.1099/ijsem.0.006799)
Supplement: Uncited Supplementary Material 1. [file ijsem-75-06799-s001.pdf]

Supplementary materials

Pseudomonas lyxosi sp. nov., *Pseudomonas arabinosi* sp. nov. and *Pseudomonas frigoris* sp. nov., isolated from glaciers

Chan Zhao, Yu-Hua Xin, and Qing Liu

Table S1. The closely relatives of strains LB3P38^{T}, LT1P18^{T} and ZB1P45^{T} based on 16S rRNA gene sequence.

Strains related to LB3P38^{T} with 16S rRNA gene sequence similarity higher than 98.6%:

Rank	Strain	Similarity (%)
1	*Pseudomonas nunensis* In5^{T} (GQ254719)	99.66
2	*Pseudomonas silesiensis* A3^{T} (KX276592)	99.52
3	*Pseudomonas piscicola* P50^{T} (LR797558)	99.51
4	*Pseudomonas mandelii* NBRC 103147^{T} (BDAF01000092)	99.45
5	*Pseudomonas svalbardensis* S025^{T} (PP478174)	99.40
6	*Pseudomonas farris* SWRI79^{T} (GCA 019145235.1 21)	99.38
7	*Pseudomonas frederiksbergensis* JAJ28^{T} (AJ249382)	99.38
8	*Pseudomonas caspiana* FBF102^{T} (LOHF01000033)	99.32
9	*Pseudomonas folii* DOAB1069^{T} (JACONW010000235)	99.25
10	*Pseudomonas mucoides* P154a^{T} (LR797589)	99.19
11	*Pseudomonas meliae* CFBP 3225^{T} (JYHE01000183)	99.18
12	*Pseudomonas ficuserectae* JCM 2400^{T} (AB021378)	99.16
13	*Pseudomonas amygdali* CFBP 3205^{T} (JYHB01000005)	99.11
14	*Pseudomonas tremae* CFBP 6111^{T} (AJ492826)	99.11
15	*Pseudomonas congelans* DSM 14939^{T} (FNJH01000022)	99.04
16	*Pseudomonas cerasi* 58^{T} (LT222319)	99.04
17	*Pseudomonas zarinae* SWRI108^{T} (JABWQZ010000068)	99.04
18	*Pseudomonas marvdashtae* SWRI102^{T} (JABWQX010000026)	99.04
19	*Pseudomonas caricapapayae* ATCC 33615^{T} (D84010)	99.03
20	*Pseudomonas iridis* P42^{T} (LR797554)	99.03
21	*Pseudomonas anatoliensis* P9^{T} (LR797545)	99.03
22	*Pseudomonas syringae* KCTC 12500^{T} (KI657453)	98.97
23	*Pseudomonas cannabina* CFBP 2341^{T} (AJ492827)	98.97
24	*Pseudomonas kilonensis* DSM 13647^{T} (LHVH01000037)	98.97
25	*Pseudomonas ogarae* F113^{T} (NC 016830)	98.97
26	*Pseudomonas thivervalensis* DSM 13194^{T} (LHVE01000021)	98.97
27	*Pseudomonas corrugata* ATCC 29736^{T} (D84012)	98.96
28	*Pseudomonas savastanoi* ATCC 13522^{T} (AB021402)	98.90
29	*Pseudomonas alvandae* SWRI17^{T} (GCA 019141525.1 1)	98.90
30	*Pseudomonas lini* CFBP 5737^{T} (AY035996)	98.90
31	*Pseudomonas kielensis* MBT-1^{T} (MW377589)	98.83
32	*Pseudomonas tehranensis* SWRI196^{T} (JABWQV010000431)	98.83
33	*Pseudomonas canavaninivorans* HB002^{T} (MZ644983)	98.83
34	*Pseudomonas zanjanensis* SWRI12^{T} (JABWRB010000017)	98.83
35	*Pseudomonas brassicacearum* ATCC 49054^{T} (EU391388)	98.83
36	*Pseudomonas marginalis* ATCC 10844^{T} (AJ308309)	98.82
37	*Pseudomonas bijieensis* L22-9^{T} (MT835388)	98.76
38	*Pseudomonas arsenicoxydans* CECT 7543^{T} (LT629705)	98.70
39	*Pseudomonas pisciculturae* P115^{T} (LR797573)	98.67
40	*Pseudomonas migulae* CIP 105470^{T} (AF074383)	98.63
**Strains related to LT1P18^{T} with 16S rRNA gene sequence similarity higher than 98.6%:**		
1	*Pseudomonas nunensis* In5^{T} (GQ254719)	99.66
2	*Pseudomonas silesiensis* A3^{T} (KX276592)	99.52
3	*Pseudomonas piscicola* P50^{T} (LR797558)	99.51
4	*Pseudomonas mandelii* NBRC 103147^{T} (BDAF01000092)	99.45
5	*Pseudomonas svalbardensis* S025^{T} (PP478174)	99.40
6	*Pseudomonas farris* SWRI79^{T} (GCA 019145235.1 21)	99.38
7	*Pseudomonas frederiksbergensis* JAJ28^{T} (AJ249382)	99.38
8	*Pseudomonas caspiana* FBF102^{T} (LOHF01000033)	99.32
9	*Pseudomonas folii* DOAB1069^{T} (JACONW010000235)	99.25
10	*Pseudomonas mucoides* P154a^{T} (LR797589)	99.19
11	*Pseudomonas meliae* CFBP 3225^{T} (JYHE01000183)	99.18
12	*Pseudomonas ficuserectae* JCM 2400^{T} (AB021378)	99.16
13	*Pseudomonas amygdali* CFBP 3205^{T} (JYHB01000005)	99.11
14	*Pseudomonas tremae* CFBP 6111^{T} (AJ492826)	99.11
15	*Pseudomonas congelans* DSM 14939^{T} (FNJH01000022)	99.04
16	*Pseudomonas cerasi* 58^{T} (LT222319)	99.04
17	*Pseudomonas marvdashtae* SWRI102^{T} (JABWQX010000026)	99.04
18	*Pseudomonas caricapapayae* ATCC 33615^{T} (D84010)	99.03
19	*Pseudomonas iridis* P42^{T} (LR797554)	99.03

20	*Pseudomonas anatoliensis* P9^{T} (LR797545)	99.03
21	*Pseudomonas syringae* KCTC 12500^{T} (KI657453)	98.97
22	*Pseudomonas cannabina* CFBP 2341^{T} (AJ492827)	98.97
23	*Pseudomonas kilonensis* DSM 13647^{T} (LHVH01000037)	98.97
24	*Pseudomonas ogarae* F113^{T} (NC 016830)	98.97
25	*Pseudomonas zarinae* SWRI108^{T} (JABWQZ010000068)	98.97
26	*Pseudomonas thivervalensis* DSM 13194^{T} (LHVE01000021)	98.97
27	*Pseudomonas corrugata* ATCC 29736^{T} (D84012)	98.96
28	*Pseudomonas savastanoi* ATCC 13522^{T} (AB021402)	98.90
29	*Pseudomonas alvandae* SWRI17^{T} (GCA 019141525.1 1)	98.90
30	*Pseudomonas marginalis* ATCC 10844^{T} (AJ308309)	98.89
31	*Pseudomonas kielensis* MBT-1^{T} (MW377589)	98.83
32	*Pseudomonas tehranensis* SWRI196^{T} (JABWQV010000431)	98.83
33	*Pseudomonas alvandae* SWRI17^{T} (MZ644983)	98.83
34	*Pseudomonas zanjanensis* SWRI12^{T} (JABWRB010000017)	98.83
35	*Pseudomonas lini* CFBP 5737^{T} (AY035996)	98.83
36	*Pseudomonas brassicacearum* ATCC 49054^{T} (EU391388)	98.83
37	*Pseudomonas bijieensis* L22-9^{T} (MT835388)	98.76
38	*Pseudomonas arsenicoxydans* CECT 7543^{T} (LT629705)	98.70
39	*Pseudomonas migulae* CIP 105470^{T} (AF074383)	98.63
40	*Pseudomonas prosekii* LMG 26867^{T} (LT629762)	98.63
**Strains related to ZB1P45^{T} with 16S rRNA gene sequence similarity higher than 98.6%:**		
1	*Pseudomonas nunensis* In5^{T} (GQ254719)	99.93
2	*Pseudomonas silesiensis* A3^{T} (KX276592)	99.79
3	*Pseudomonas mandelii* NBRC 103147^{T} (BDAF01000092)	99.73
4	*Pseudomonas farris* SWRI79^{T} (GCA 019145235.1 21)	99.73
5	*Pseudomonas svalbardensis* S025^{T} (PP478174)	99.73
6	*Pseudomonas frederiksbergensis* JAJ28^{T} (AJ249382)	99.73
7	*Pseudomonas piscicola* P50^{T} (LR797558)	99.59
8	*Pseudomonas caspiana* FBF102^{T} (LOHF01000033)	99.59
9	*Pseudomonas folii* DOAB1069^{T} (JACONW010000235)	99.52
10	*Pseudomonas meliae* CFBP 3225^{T} (JYHE01000183)	99.45
11	*Pseudomonas amygdali* CFBP 3205^{T} (JYHB01000005)	99.38
12	*Pseudomonas tremae* CFBP 6111^{T} (AJ492826)	99.38
13	*Pseudomonas ficuserectae* JCM 2400^{T} (AB021378)	99.37
14	*Pseudomonas cannabina* CFBP 2341^{T} (AJ492827)	99.25
15	*Pseudomonas congelans* DSM 14939^{T} (FNJH01000022)	99.25
16	*Pseudomonas cerasi* 58^{T} (LT222319)	99.25
17	*Pseudomonas caricapapayae* ATCC 33615^{T} (D84010)	99.24
18	*Pseudomonas syringae* KCTC 12500^{T} (KI657453)	99.18
19	*Pseudomonas savastanoi* ATCC 13522^{T} (AB021402)	99.18
20	*Pseudomonas iridis* P42^{T} (LR797554)	99.11
21	*Pseudomonas anatoliensis* P9^{T} (LR797545)	99.11
22	*Pseudomonas kilonensis* DSM 13647^{T} (LHVH01000037)	99.04
23	*Pseudomonas ogarae* F113^{T} (NC 016830)	99.04
24	*Pseudomonas lini* CFBP 5737^{T} (AY035996)	99.04
25	*Pseudomonas marginalis* ATCC 10844^{T} (AJ308309)	99.04
26	*Pseudomonas arsenicoxydans* CECT 7543^{T} (LT629705)	98.97
27	*Pseudomonas zarinae* SWRI108^{T} (JABWQZ010000068)	98.97
28	*Pseudomonas corrugata* ATCC 29736^{T} (D84012)	98.96
29	*Pseudomonas prosekii* LMG 26867^{T} (LT629762)	98.90
30	*Pseudomonas alvandae* SWRI17^{T} (GCA 019141525.1 1)	98.90
31	*Pseudomonas mucoides* P154a^{T} (LR797589)	98.86
32	*Pseudomonas tehranensis* SWRI196^{T} (JABWQV010000431)	98.83
33	*Pseudomonas alvandae* SWRI17^{T} (MZ644983)	98.83
34	*Pseudomonas zanjanensis* SWRI12^{T} (JABWRB010000017)	98.83
35	*Pseudomonas brassicacearum* ATCC 49054^{T} (EU391388)	98.83
36	*Pseudomonas pisciculturae* P115^{T} (LR797573)	98.83
37	*Pseudomonas chlororaphis subsp. chlororaphis* NBRC 3904^{T} (BCZX01000031)	98.77
38	*Pseudomonas viciae* 11K1^{T} (MN698727)	98.77
39	*Pseudomonas marvdashtae* SWRI102^{T} (JABWQX010000026)	98.77
40	*Pseudomonas thivervalensis* DSM 13194^{T} (LHVE01000021)	98.76
41	*Pseudomonas bijieensis* L22-9^{T} (MT835388)	98.76

Table S2. The genome information of strains LB3P38^{T}, LT1P18^{T} and ZB1P45^{T}.

	LB3P38^{T}	LT1P18^{T}	ZB1P45^{T}
**Size (Mb)**	6.72	6.93	6.80
**GC (%)**	58.9	58.8	58.7
**No. of contigs**	1	1	1
**N50 (bp)**	6,722,390	6,932,784	6,805,123
**No. of**			
protein-coding genes	6,047	6,291	6,174
pseudogenes	13	20	34
tRNA	71	73	70
5S rRNA	8	8	8
16S rRNA	7	7	7
23S rRNA	7	7	7
ncRNA	59	61	71
tmRNA	1	1	1

Table S3. The pairwise ANI (lower right 'triangle') and dDDH (upper right 'triangle') values (%) between the three strains and their closest relatives.

Strains: 1, LB3P38^{T}; 2, LT1P18^{T}; 3, ZB1P45^{T}; 4, *P. svalbardensis* CCTCC AB 2023225^{T}; 5, *P. frederiksbergensis* LMG 19851^{T}. The results were based on formula d_4 as implemented by TYGS.

	1	2	3	4	5
1	-	59.4	61.0	59.4	52.8
2	94.3	-	57.7	57.9	53.1
3	94.6	94.2	-	58.5	52.1
4	94.4	94.4	94.3	-	63.4
5	93.1	93.2	93.0	93.2	-

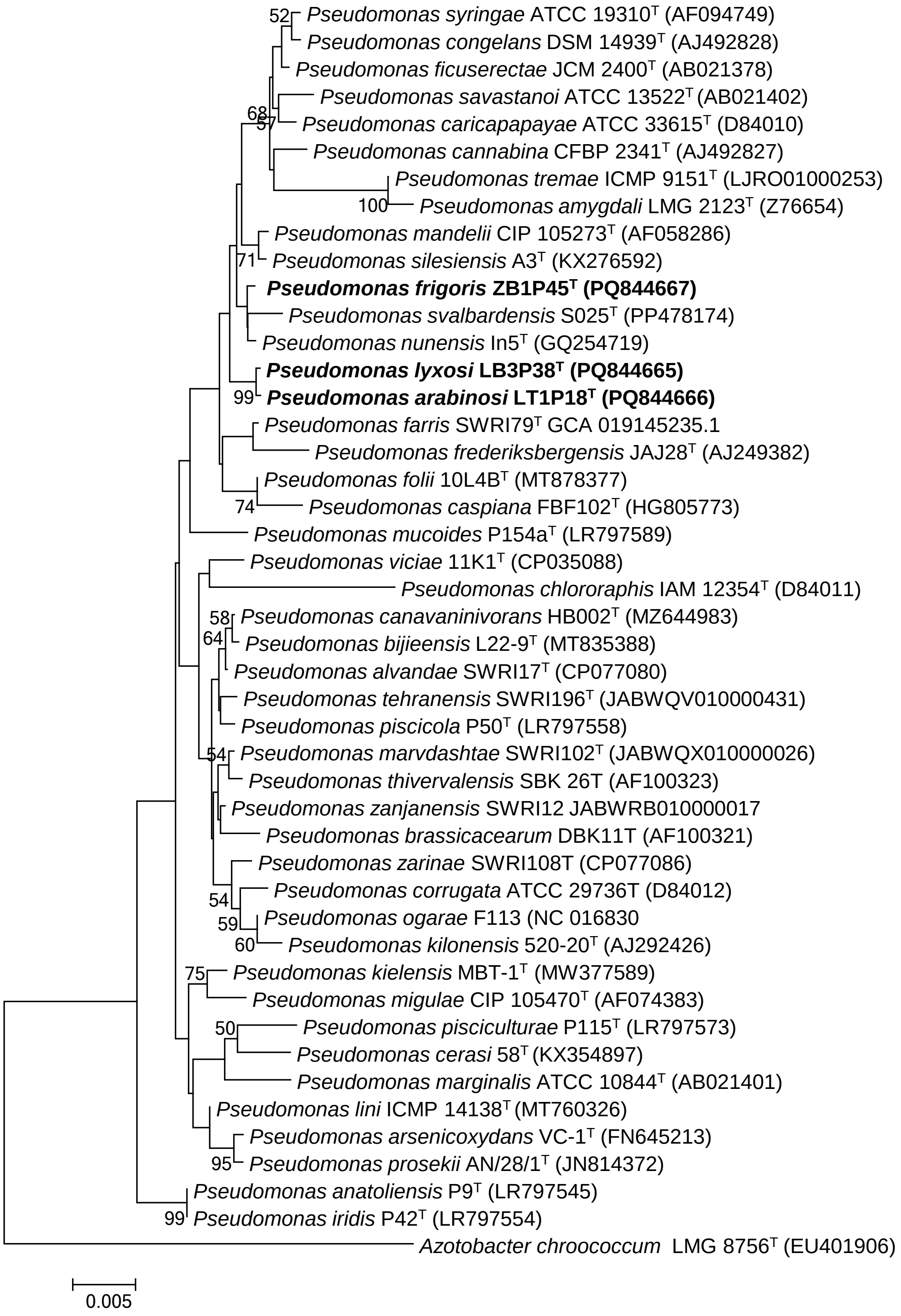

Fig. S1. Phylogenetic tree of strains LB3P38^{T}, LT1P18^{T}, ZB1P45^{T}, and related strains based on the 16S rRNA gene sequence comparisons using the NJ method. GenBank accession numbers of the 16S rRNA gene sequences are given in parentheses. *Azotobacter chroococcum* LMG 8756^{T} was used as an outgroup. Bootstrap values (>50 %) based on 1,000 replicates are shown at the branch nodes. Bar, 0.005 substitutions per nucleotide positions.

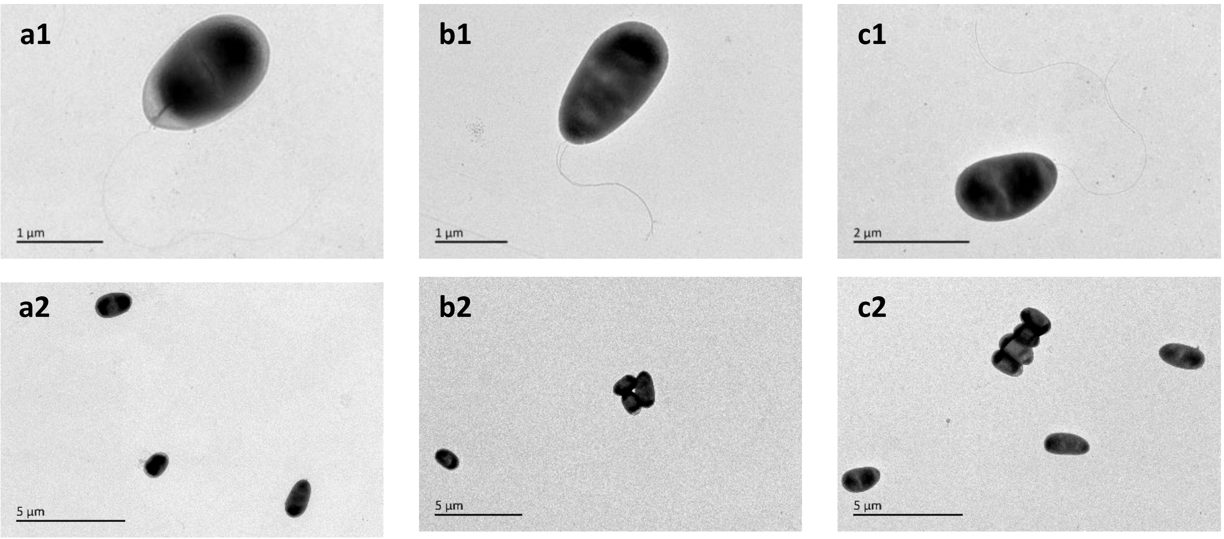

Fig. S2. Transmission electron micrograph of strains LB3P38^{T} (a1,a2), LT1P18^{T} (b1,b2), and ZB1P45^{T} (c1,c2) grown at 30°C on NB agar.
